# Cost-effectiveness analysis of oral anti-viral drugs used for treatment of chronic hepatitis B in Turkey

**DOI:** 10.1186/s12962-015-0046-8

**Published:** 2015-12-10

**Authors:** Guvenc Kockaya, Akin Kose, Fatma Betul Yenilmez, Oktay Ozdemir, Ece Kucuksayrac

**Affiliations:** Gilead Sciences Turkey, Istanbul, Turkey; Akıl Consultancy, Ankara, Turkey; Yorum Consultancy, Istanbul, Turkey

**Keywords:** Chronic hepatitis B, Cost effectiveness, Oral antiviral treatment

## Abstract

**Background:**

All international guidelines suggested that Tenofovir and Entecavir are the primary drugs at the first line therapy for the treatment of chronic hepatitis B (CHB). However, in Turkey these medications reimbursed at the second line therapy according to the Healthcare Implementation Notification. The aim of this study is to compare the cost effectiveness of oral antiviral treatment strategies in CHB for Turkey using lamuvidine, telbuvidine, entecavir, and tenofovir as medications.

**Methods:**

The analysis was conducted using Markov models. The analysis scenarios based on first line treatment options with Lamuvidine, Telbuvidine, Entecavir, and Tenofovir as the medications. In the analysis, inadequate response or resistance after receiving 12 months of the treatment with Entecavir and Telbivudine were compared to the results found from switching from Entecavir to Tenofovir or from switching from Telbuvidine to Tenofovir. In additional, inadequate response or resistance after receiving 6 months of the treatment for Lamivudine was compared to the results found from switching from Lamivudine to Tenofovir. The study population included men and women, who were 40 years of age. The patients` compliance was estimated 100 % for all of the therapy options. The model duration was constructed to evaluate, treatment strategy duration of 40 years. The cost of medications, examinations/follow-ups and complications were included in the model. Years of Potential Life Lost was used as the health outcome. An incremental cost-effectiveness ratio analysis has been conducted.

**Results and discussion:**

While the minimum years of life lost was found as 0.22 with tenofovir treatment in 5 years, treatment cost was calculated as 12,169 TL. These values were detected as 0.56 years and 7727 TL, 0.37 years and 12,770 TL, respectively for lamuvidine and telbuvidine treatments. The maximum years of life lost and treatment cost was with lamuvidine treatment were detected as 1.60 years and 18,813 TL and, secondly 0.89 years and 24,007 TL for lamuvidine-tenofovir treatment during 10 years. The minimum years of life lost and cost are 0.54 year and 35,821 TL for tenofovir treatment during 10 years. The minimum years of life lost and cost were determined as 1.21 years and 52,839 TL for tenofovir treatment strategy during 20 years. During 30 years period, tenofovir treatment was found to have the minimum years of life lost (1.73 years) and minimum cost (84,149 TL). When the results of 40 years period were analyzed, years of life lost and costs are 2.06 years and 119,604 TL, 2.13 years and 162,115 TL, 2.13 years and 161,642 TL, 6.52 years and 147,245 TL, 3.20 years and 132,157 TL, 4.10 years and 151,059 TL and 3.05 years and 138,182 TL for tenofovir, entecavir, entecavir-tenofovir, lamuvidine, lamuvidine-tenofovir, telbivudine and telbivudine-tenofovir.

**Conclusions:**

In the model presented in this study, in cost effectiveness analysis about CHB treatments, Tenofovir was found to be one of the cost effective methods in comparison with other treatment strategies different time intervals. Beyond this achievement Tenofovir has shown to reduce cumulative treatment cost in first line CHB treatment when compared with regard to 40 year cumulative treatment cost.

## Background

Hepatitis B is among the most common infectious diseases worldwide. At least 2 billion individuals are estimated to have been infected with the Hepatitis B virus (HBV), and 378 million individuals (6 % of the world’s population) are estimated to be chronic carriers worldwide. Cirrhosis, hepatic failure, or hepatocellular cancer are known to develop in approximately 40 % of all chronic hepatitis B (CHB) cases [1, 2].

The prevalence of the hepatitis B surface antigen (HBsAg) was determined as 4.57 % according to a meta-analysis conducted to investigate HBV epidemiology in Turkey. The confidence interval (CI) was determined as 3.58–5.76, and the estimated number of CHB cases was 3.3 million. This ratio increases to 9.8 % in the eastern part of Turkey. According to the epidemiologic data obtained between 1999 and 2009, while Hepatitis B prevalence is 3.23 % in the age group of 0–14 years, it is 5.77 % in the age group of 15–24 years. This ratio is 7.08 % in the 25–34 age group, 6.93 % in the 35–44 age group, 6.13 % in the 45–54 age group, and 5.02 % in the 55–64 age group. Hepatitis B surface antigen (HBsAg) prevalence increases with age according to this study [3].

Oral antiviral treatment is the most convenient way to control and stabilize the CHB disease. The indications are generally the same for HBeAg positive and negative patients [4]. Treatment is usually based on assessing the combination of three criteria: serum HBV DNA values, ALT values, and the stage of the hepatic disease [4].

The goal of treatment for Chronic Hepatitis B is to prevent the progression to cirrhosis, reducing the need for liver transplantation and improving the quality of life of the patient. Both national and international CHB treatment guidelines reveal that HBV DNA should be strongly suppressed for achieving these goals. With the treatment, the histopathological findings of the liver and the biochemical parameters are improved and the long-term complications of CBH are reduced as well [4].

Some current treatment guidelines, such as the European Association for the Study of the Liver (EASL), the American Association for the Study of Liver Diseases (AASLD) and the Asia Pacific Association for the Study of the Liver (APASL) recommend potent Oral Anti-viral (OAV) treatments as the first choice in initial therapy for achieving the specified goals [4–6]. In Turkey, changes have been made in Healthcare Reimbursement Rules to be aligned with International Hepatology Management and Treatment guidelines. Potent OAV treatments, such as Tenofovir and Entecavir, were approved to be used for a low viral loaded patient population in 2014 [8].

Tenofovir maintains the effective suppression of HBV DNA through 8 years of treatment with no evidence of TDF resistance in contrast to other agents [8]. In addition to the effective suppression of HBV DNA with a TDF treatment, there have been histological improvements in a 5-year treatment both in cirrhotic and non-cirrhotic CHB patients [9]. As Entecavir has a 1.2 % resistance potential in a naïve treatment group, Entecavir patients should also be closely monitored for resistance. In addition, in long-term use, the cumulative probability of Entecavir resistance development in lamivudine-refractory patients was reported to be as high as 57 % over 6 years of treatment [11]. In a 2-year Globe trial, it was shown that the Telbivudine resistance rates were 25.1 % for HBeAg positive patients and 10.8 % for HBeAg negative patients. The M204I signature mutation was the primary basis for Telbivudine resistance, with secondary mutations detected at the L80, L180, and other codons. In vitro studies have shown that HBV with the M204I mutation remains sensitive to the nucleotide analogues adefovir, dipivoxil, and tenofovir [7].

Lamivudine has been approved in Turkey since 2000. For a long period of time, lamivudine was the only treatment before Entecavir approval in 2006. Tenofovir and Telbivudine were approved in Turkey in 2007 and 2008, respectively. Based on the Globe [7] study’s results, a road map concept has been generated by the Social Security Institute [10]. From 2009 to 2015, Lamivudine and Telbivudine was stratified as the first line treatment options for low viral loaded patients (HBV DNA <104 IU/mL). As a result, 2/3 of HBV patients had been treated with Lamivudine and Telbivudine. Tenofovir was only prescribed for high viral loaded patients (HBV DNA >104 IU/mL) or patients with a detectable HBV DNA level after 6 months of therapy with Lamivudine or Telbivudine.

This analysis compares Lamivudine, Telbivudine, Entecavir, and Tenofovir, the oral antiviral agents used for first line CHB treatment according to the Health Application Notification, which defined the reimbursement process in Turkey.

## Methods

### Treatment strategies

**Fig. 1:**
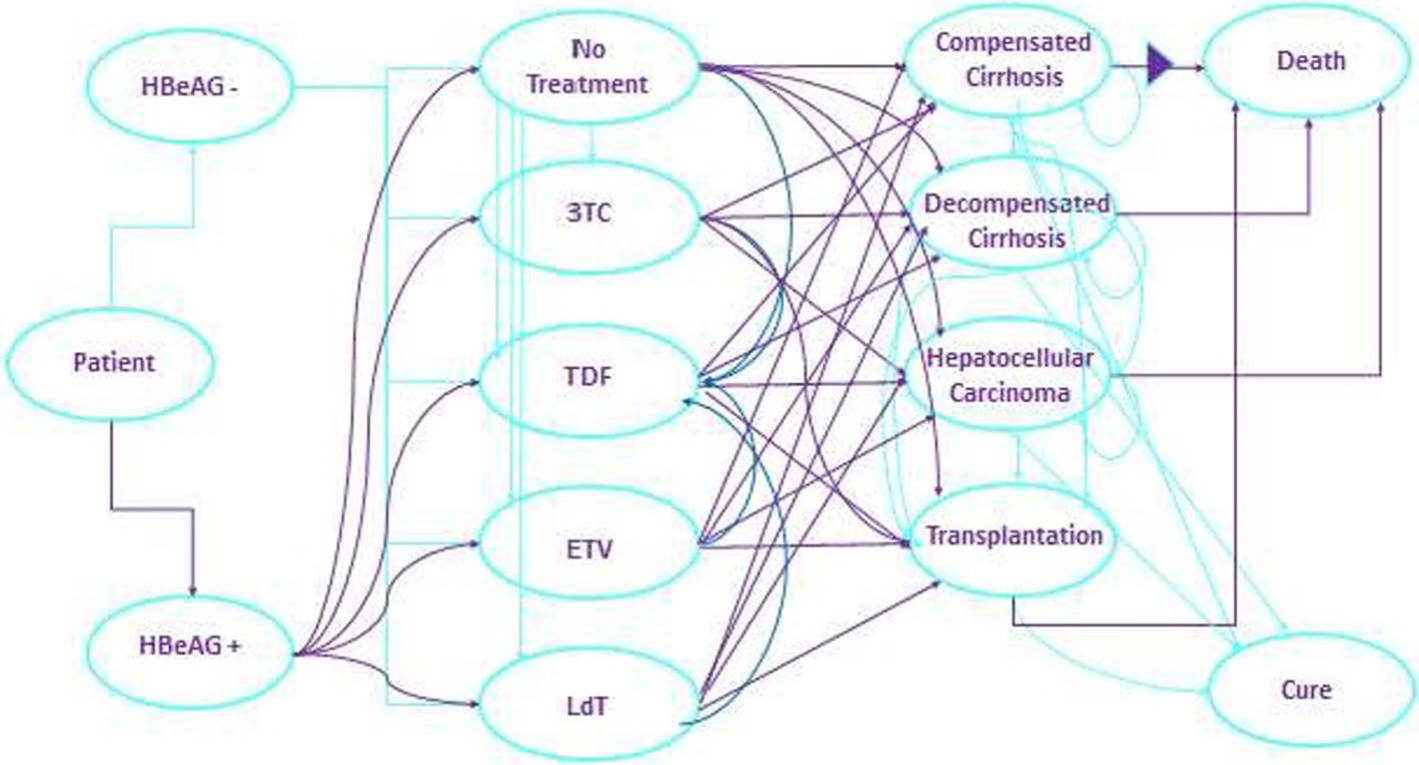
Chronic hepatitis B treatment Markov model

The Markov model was used in the present analysis. The Markov model is designed to include the entire disease whether the patients respond to therapy or not, complications develop in the case of irresponsiveness to therapy, complications progress in years, and a follow up for the patients until death (Fig. 1). In the analysis scenarios, Lamivudine (3TC), Telbivudine (LdT), Entecavir (ETV), and Tenofovir (TDF) use was planned for first line therapy. Treatments that switched to Tenofovir from Entecavir (ETV- TDF) and to Tenofovir from Telbivudine (LdT-TDF) in the case of an inadequate response or resistance development after 12 months or that switched to Tenofovir from Lamivudine (3TC-TDF) in the case of an inadequate response or responsiveness 6 months later were included in the analysis. A single strategy was also included during the model time in all molecules in transitional treatments. The resistance rates of the drugs and the estimated 5-year virologic response rates for each treatment were obtained from published studies.

### Treatment population

The age for beginning treatment was 40 years, and the duration of treatment was 40 years, which is consistent with the life expectancy. Of the patients, 50 % were assumed to be male and 50 % were assumed to be female in the Turkish population [8]. They were assumed to be 100 % compliant to therapy. The HBeAg positivity rate was assumed to be 30 % [9]. The transition probability between disease stages was taken from the analysis of Kanwal et al. [13]. (Table 1). Tables 2 and 3 show the response rates of CHB treatments in the model and the resistance rates. The rates of resistance developing during the therapy and the cumulative resistance rates for the first 3 years of therapy were obtained from the data of the paper published by Marcellin et al., and the assumptions were used for the resistance rates after 3 years [11, 12]. The resistance data of Entecavir treatment for the first 6 years were obtained from the study published by Tenney et al., and the assumptions were made for the following years [14]. The Lamivudine resistance data were calculated proportionally with the yearly resistance rates of the patients who had a high viral load for the fourth and the fifth years, and it was assumed to be half of the previous year for each year after the fifth year [15]. The Telbuvidin response data were obtained from the data of Zeuzem et al. and calculated proportionally with 3TC treatment at the third year and thereafter [16] (Table. 2, 3).

**Table 1:** Transition rates between chronic hepatitis B and diseases [13]

	Decompensated cirrhosis	Hepatocellular cancer	Transplantation	Death (%)
Compensated cirrhosis	7.3 %	3.4 %	0.0 %	4.9
Decompensated cirrhosis	–	3.4 %	20.0 %	19.0
Hepatocellular cancer	–	–	25.0 %	43.3
transplantation	–	–	–	6.9

**Table 2:** Response rates to chronic hepatitis B treatments [17–20]

24th week	TDF	ETV	3TC	LAM	LdT	TDF	ETV	3TC	LAM	LdT
<300	84.8	84.8	73.0	71.0	80.0	48.9	37.0	34.0	32.0	45.0
300–104	9.7	9.7	15.0	20.0	15.0	32.5	40.0	26.0	31.0	31.0
104–105	2.7	2.7	3.6	2.7	1.5	8.9	11.0	12.0	11.1	7.2
105–106	0.2	0.2	1.5	1.1	0.6	0.8	1.0	5.0	4.6	3.0
>106	2.7	2.7	6.9	5.2	2.9	8.9	11.0	23.0	21.3	13.8
48th week										
<300	93.2	93.3	75.6	71.4	88.3	76.1	69.1	39.8	40.4	60.0
300–104	4.2	4.1	12.5	18.4	8.0	19.1	24.7	18.2	28.1	21.5
104–105	1.6	1.6	5.1	7.5	3.3	3.4	4.4	11.7	18.1	13.8
105–106	0.3	0.3	2.0	0.8	0.1	0.5	0.6	9.3	4.1	1.4
>106	0.6	0.6	4.8	1.9	0.3	0.9	1.2	21.0	9.3	3.3

Values are expressed as %

**Table 3:** Resistance to therapy for chronic Hepatitis B treatment [11–16]

Years	1	2	3	4	5	6	7	8	9	10	11	12	13	14	15	16	17	18	19
Resistance																			
TDF	0.0	0.0	0.0	0.0	0.0	0.0	0.0	0.0	0.0	0.0	0.0	0.0	0.0	0.0	0.0	0.0	0.0	0.0	0.0
ETV	0.2	0.3	0.7	0.0	0.0	0.0	0.0	0.0	0.0	0.0	0.0	0.0	0.0	0.0	0.0	0.0	0.0	0.0	0.0
3TC	6.7	11.3	21.0	11.3	6.4	3.2	1.6	0.8	0.4	0.2	0.1	0.0	0.0	0.0	0.0	0.0	0.0	0.0	0.0
LtD/Hbe−	1.1	2.2	4.1	2.2	1.2	0.6	0.3	0.2	0.1	0.0	0.0	0.0	0.0	0.0	0.0	0.0	0.0	0.0	0.0
LtD/Hbe+	5.0	6.3	11.6	6.3	3.5	1.8	0.9	0.4	0.2	0.1	0.1	0.0	0.0	0.0	0.0	0.0	0.0	0.0	0.0
Cumulative resistance																			
TDF	0.0	0.0	0.0	0.0	0.0	0.0	0.0	0.0	0.0	0.0	0.0	0.0	0.0	0.0	0.0	0.0	0.0	0.0	0.0
ETV	0.2	0.5	1.2	1.2	1.2	1.2	1.2	1.2	1.2	1.2	1.2	1.2	1.2	1.2	1.2	1.2	1.2	1.2	1.2
3TC	6.7	18.0	39.0	50.3	56.7	59.9	61.5	62.3	62.7	62.9	63.0	63.1	63.1	63.1	63.1	63.1	63.1	63.1	63.1
LtD/Hbe−	1.1	3.3	7.4	9.6	10.8	11.4	11.8	11.9	12.0	12.0	12.1	12.1	12.1	12.1	12.1	12.1	12.1	12.1	12.1
LtD/Hbe +	5.0	11.3	22.9	29.1	32.7	34.4	35.3	35.8	36.0	36.1	36.1	36.2	36.2	36.2	36.2	36.2	36.2	36.2	36.2

Values are expressed as %

The rates of response developing during therapy and the data were obtained from the paper published by Marcellin et al., and the assumptions were used for the response rates for 48 weeks of therapy for Tenofovir [17]. The response data of Entecavir treatment for 48 weeks were obtained from the study published by Arnold et al. [18]. The Lamivudine response data were obtained from the publications of Yuan et al. and Lai et al. [19, 20]. The Telbuvidin resistance data were obtained from the data of Lai et al. All assumptions and selected articles for modeling were based on three experts’ opinions.

### Cost calculation

The cumulative treatment cost was calculated by including the costs of medicines, tests/follow ups, and complications. The costs paid by the Social Security Institution (SSI) on September 1, 2014 were taken for the calculation of the costs of medicines and tests/follow ups. The average annual costs of the treatment strategies included in the model according to the costs paid by the published figures are shown in Table 4. An annual 3 % discount rate was used for the calculation of the costs over the 40-year model process [21]. It was assumed that the discount rate covered the inflator and deflator effect.

**Table 4:** Treatment strategies for chronic hepatitis B in Turkey and the annual treatment costs

Medicines	Dose	Unit	Dose (mg/tablet)	Package (tablet/box)	The average annual public
					Cost of treatment (TL)
3TC	100	mg/day	100	28	839.94
ETV	0.5	mg/kg/day	0.5	30	3497.53
TDF	300	mg/day	300	30	2236.42
LdT	600	mg/day	600	28	2033.61

The complication costs were taken from the study published in 2009 [22, 23]. The data of 2014 were obtained by adapting the 2009 costs to a 3 % discount rate [21] (Table 5). The transition risks according to the plasma HBV DNA levels and the inter-transition of the complications and the mortality rates were taken from the published studies in the application of the complication costs to the treatment strategies [11–17, 19, 24–26].

**Table 5:** The average annual costs of disease conditions in Turkey

Disease states	Original costs (TL/year)	
Year	2009 (TL)	2014^a^ (TL)
Compensated cirrhosis	6778	7857
Decompensated cirrhosis	7573	8780
Hepatocellular carcinoma	31,044	35,989

^a^2014 costs were calculated by using 3 % reduction ratio according to 2009

### Calculation of clinical effectiveness

Years of life lost (YLL) was used for the clinical effectiveness parameter. The life expectancy of healthy individuals according to the life expectancy at birth in Turkey was used for the response to therapy, and it was calculated again for each year in the model [27]. In the case of irresponsiveness to therapy, published complications and mortality transitions were used for the calculation of the YLL, and it was calculated again for each year in the model [11, 12, 14, 16, 19, 22–26].

### Comparison of overall cost and clinical effectiveness

The calculated cumulative treatment cost and clinical effectiveness outcomes were compared according to 5, 10, 20, 30, and 40 years of data calculated in the model.

### Comparison of cost-effectiveness

The incremental cost-effectiveness ratio (ICER) was used for the comparison of cost-effectiveness. The costs were sorted from minimum to maximum for the ICER calculation. An assessment was performed between the second leading minimum cost and the maximum cost. If a treatment cost was lower than the next treatment cost and its effectiveness was higher, it was stated to be “superior,” and the assessment was performed with the next treatment. If the effectiveness was high along with a high cost, the ICER formulation was applied as follows [27]:

$${\text{ICER}} = \frac{{{\text{ Total Cost}_{\text {Anti-Viral 1}}}{-}{\text{ Total Cost}_{\text {Anti-Viral 2}}}}}{{{\text{Clinical effectiveness}_{\text {Anti-Viral 1}}} {-}{\text{ Clinical Effectiveness}_{\text {Anti-Viral 2}}}}}$$

The cumulative treatment cost (medicines, tests/follow ups, and complications) was calculated at the end of 40 years, which is the model year, and the YLL was used for the comparison of cost effectiveness.

### Sensitivity analysis

The sensitivity analysis was conducted to show the impact of the change of the parameters on the cost-effectiveness ratio. A 25 % increase and decrease of the inputs method was used for the analysis [28].

## Results and discussion

The treatment strategies were determined and analyzed for Tenofovir (TDF), Entecavir (ETV), switching to Tenofovir from Entecavir (ETV-TDF), Lamivudine (3TC), switching to Tenofovir from Lamivudine (3TC-TDF), Telbivudine (LdT), and switching to Tenofovir from Telbivudine (LdT-TDF).

### Comparison of overall cost and clinical effectiveness

While the minimum years of life lost was found to be 0.22 with TDF treatment for 5 years, the treatment cost was calculated as 12,169 TL. These values were identified as 0.56 years and 7727 TL and 0.37 years and 12,770 TL, respectively, for the 3TC and LdT treatments.

The maximum years of life lost and the treatment cost with the 3TC treatment were identified as 1.60 years and 18,813 TL, and, secondly, 0.89 years and 24,007 TL for the 3TC-TDF treatment for 10 years. The years of life lost and the treatment costs are 0.78 years and 26,848 TL, 1.01 years and 27,295 TL, 0.57 years and 35,918 TL, 0.57 years and 35,918 TL for the other treatment alternatives LdT-TDF, LdT, ETV, and ETV-TDF, respectively. The minimum years of life lost and cost are 0.54 year and 35,821 TL for the TDF treatment for 10 years (Table 6).

**Table 6:** Years of life lost and treatment costs for each 10 years per capita

Treatments	Year 10		Year 20		Year 30		Year 40	
	YLL	Total cost (TL)	YLL	Total cost (TL)	YLL	Total cost (TL)	YLL	Total cost (TL)
TDF	0.54	24,948	1.21	52,839	1.73	84,149	2.06	119,604
3TC-TDF	0.89	24,007	1.91	54,785	2.70	90,517	3.20	132,157
LdT-TDF	0.78	26,848	1.77	58,542	2.56	95,357	3.05	138,182
3TC	1.60	18,813	3.81	49,974	5.50	92,666	6.52	147,245
LdT	1.01	27,295	2.37	61,448	3.44	102,458	4.10	151,059
ETV-TDF	0.57	35,821	1.25	74,366	1.79	116,074	2.13	161,642
ETV	0.57	35,918	1.25	74,592	1.79	116,425	2.13	162,115

The minimum years of life lost and the cost were determined as 1.21 years and 52,839 TL for the TDF treatment strategy for 20 years. The years of life lost and the cost for the other treatment strategies, ETV, ETV-TDF, LdT-TDF, 3TC-TDF, LdT, and 3TC, are 1.25 years and 74,592 TL, 1.25 years and 74,367 TL, 1.77 years and 58,542 TL, 1.91 years and 54,785 TL, 2.37 years and 61,448 TL, and 3.81 years and 49,974 TL, respectively.

Over a 30-year period, the TDF treatment was found to have the minimum years of life lost (1.73 years) and the minimum cost (84,149 TL). The years of life lost and the treatment costs are 1.79 years and 116,425 TL for ETV, 1.79 years and 116,074 TL for ETV-TDF, 5.50 years and 92,666 TL for 3TC, 2.70 years and 90,517 TL for 3TC-TDF, 3.44 years and 102,458 TL for LdT, and 2.56 years and 95.357 T for LdT-TDF (Table 6).

When the results of the 40-year period were analyzed, the years of life lost and costs are 2.06 years and 119,604 TL, 2.13 years and 162,115 TL, 2.13 years and 161,642 TL, 6.52 years and 147,245 TL, 3.20 years and 132,157 TL, 4.10 years and 151,059 TL, and 3.05 years and 138,182 TL for TDF, ETV, ETV-TDF, 3TC, 3TC-TDF, LdT, and LdT-TDF, respectively (Table 6).

### Comparison of cost-effectiveness

According to the 40-year treatment model designed for an incremental cost-effectiveness comparison, the TDF treatment dominated the alternative treatment strategies, as it provided the minimum total cost and the minimum years of life lost. In other words, TDF was determined to be the most cost-effective treatment strategy compared to the other treatment strategies (Table 7).

**Table 7:** Incremental cost effectiveness analysis of chronic hepatitis B treatments

Treatments	Total cost (TL)	YLL	Cost difference (TL)	Year difference	ICER
TDF	119,604	2.06			
ETV-TDF	161,642	2.13	42,037	0.07	Dominated
ETV	162,115	2.13	42,511	0.07	Dominated
LdT-TDF	138,182	3.05	18,577	0.99	Dominated
3TC-TDF	132,157	3.20	12,552	1.14	Dominated
LdT	151,059	4.10	31,455	2.04	Dominated
3TC	147,245	6.52	27,640	4.56	Dominated

Calculations were done according to the constructed 40-year treatment model

In Table 6, the minimum treatment cost belongs to TDF with 119,604 TL in the total cost and drug cost comparison. The drug cost is 84,885 TL for the TDF treatment. The total treatment cost is 147,244 TL, and the drug cost is 28,151 TL for the 3TC treatment.

### Sensitivity analysis

The sensitivity analysis was conducted for 25 % varying for each parameters. The most effective parameter for cost-effectiveness was observed as a virologic respond.

In all scenarios of the sensitivity analysis, it was shown that TDF has the lowest cost and the highest effectiveness. Based on these results, TDF dominated all options in all scenarios (Tables 8, 9).

**Table 8:** 25 % varying impact on TDF outcomes

	Age	HBeAg (−) rate (%)	TDF: virologic response 48th week in HBeAg (−) (%)	TDF: virologic response 48th week in HBeAg (+) (%)	TDF annual drug cost (TL)
Base case	40	70	93.2	76.1	2236
25 % increase	50	88	100.0	95.2	2796
25 % decrease	30	53	69.9	57.1	1677

**Table 9:** Sensitivity analysis results for the impact of each parameter on cost-effectiveness

Parameter	Change	New value		Total cost (TL)	YLL	Cost difference (TL)	Year difference	
Age = 50 years		50	TDF	119,604	1.302			
			ETV-TDF	161,642	1.347	42,037	0.045	
			ETV	162,115	1.347	42,511	0.046	
			LdT-TDF	138,182	1.923	18,577	0.622	
			3TC-TDF	132,157	2.028	12,552	0.726	
			LdT	151,059	2.581	31,455	1.280	
			3TC	147,245	4.109	27,640	2.807	
			TDF	119,604	2.987			
			ETV-TDF	161,642	3.088	42,037	0.101	
			ETV	162,115	3.089	42,511	0.102	
Age = 30 years		30	LdT-TDF	138,182	4.429	18,577	1.442	
			3TC-TDF	132,157	4.621	12,552	1.634	
			LdT	151,059	5.954	31,455	2.967	
			3TC	147,245	9.453	27,640	6.465	
			TDF	119,411	2.025			
			ETV-TDF	161,353	2.079	41,943	0.054	
			ETV	161,826	2.079	42,416	0.055	
HBeAg (−) rate	25 % increase	88 %	LdT-TDF	132,215	2.656	12,804	0.631	
			3TC-TDF	127,731	2.907	8320	0.882	
			LdT	137,643	3.152	18,233	1.128	
			3TC	128,004	5.124	8593	3.099	
			TDF	119,798	2.096			
			ETV-TDF	161,930	2.183	42,132	0.087	
			ETV	162,404	2.183	42,606	0.088	
HBeAg (−) rate	25 % decrease	53 %	LdT-TDF	144,149	3.445	24,351	1.349	
			3TC-TDF	136,582	3.487	16,784	1.391	
			LdT	164,475	5.045	44,677	2.949	
			3TC	166,486	7.907	46,688	5.811	
			TDF	119,573	2.055			
			ETV-TDF	161,642	2.131	42,068	0.076	
			ETV	162,115	2.131	42,542	0.076	
TDF: virologic response 48th week in HBeAg (−)	25 % increase	100.0 %	LdT-TDF	138,176	3.049	18,603	0.994	
			3TC-TDF	132,148	3.196	12,574	1.140	
			LdT	151,059	4.099	31,486	2.043	
			3TC	147,245	6.515	27,672	4.460	
			TDF	119,711	2.078			
			ETV-TDF	161,642	2.131	41,931	0.053	
			ETV	162,115	2.131	42,404	0.053	
TDF: Virologic response 48th week in HBeAg (−)	25 % decrease	69.9 %	LdT-TDF	138,202	3.054	18,491	0.976	
			3TC-TDF	132,186	3.202	12,475	1.124	
			LdT	151,059	4.099	31,348	2.021	
			3TC	147,245	6.515	27,534	4.437	
			TDF	119,538	2.049			
			ETV-TDF	161,642	2.131	42,103	0.082	
			ETV	162,115	2.131	42,577	0.082	
TDF: Virologic response 48th week in HBeAg (+)	25 % increase	95.2 %	LdT-TDF	138,153	3.044	18,614	0.996	
			3TC-TDF	132,118	3.190	12,580	1.141	
			LdT	151,059	4.099	31,521	2.050	
			3TC	147,245	6.515	27,707	4.467	
			TDF	119,671	2.072			
			ETV-TDF	161,642	2.131	41,971	0.059	
			ETV	162,115	2.131	42,444	0.060	
TDF: Virologic response 48th week in HBeAg (+)	25 % decrease	57.1 %	LdT-TDF	138,211	3.056	18,540	0.985	
			3TC-TDF	132,195	3.204	12,524	1.133	
			LdT	151,059	4.099	31,388	2.027	
			3TC	147,245	6.515	27,574	4.444	
			TDF	140,826	2.060			
			ETV-TDF	161,879	2.131	1053	0.070	
			ETV	162,115	2.131	1289	0.071	
TDF annual drug cost	25 % decrease	2796 TL	LdT-TDF	144,943	3.050	4117	0.990	
			3TC-TDF	147,174	3.197	6348	1.137	
			LdT	151,059	4.099	0233	2.038	
			3TC	147,245	6.515	6419	4.455	
			TDF	98,383	2.060			
			ETV-TDF	161,405	2.131	63,022	0.070	
			ETV	162,115	2.131	63,732	0.071	
TDF annual drug cost	25 % decrease	1677 TL	LdT-TDF	131,421	3.050	33,038	0.990	
			3TC-TDF	117,139	3.197	18,756	1.137	
			LdT	151,059	4.099	52,676	2.038	
			3TC	147,245	6.515	48,862	4.455	

Tenofovir dominates all comparisons in all scenarios

## Conclusions

Currently, CHB treatment has achieved positive results regarding a high virologic response and a low resistance [4]. All published current guidelines recommend Tenofovir and Entecavir as the first drug for CHB treatment.

In the study conducted in Taiwan and published by Veenstra et al. [29], a life-long Markov model was used, and Entecavir monotherapy was found to be more cost-effective compared to Lamivudine and Adefovir disoproxil combination treatments. In this analysis, Tenofovir monotherapy was found to be the most cost-effective treatment during the first 5 years, the first 10 years, for 30 years, and for 40-year life spans, which is similar to the results of the life span assessment.

In a disease burden study about HBV conducted in Vietnam and published in 2008, the average annual HBV disease cost was determined as $450.35 for this country [30]. In research conducted in China and published in 2013, the cost of CHB was found to be $4.136 [31]. In another study conducted in the US, the average life span cost per capita of HBV was estimated as $2.667 [32]. In a cost analysis of immigrants using the 2006 data in Canada, the treatment costs of Entecavir, Tenofovir, Lamivudine, and *pegylated interferon* (48 weeks) were determined as $6.504, $5.032, $1.516, and $10.185, respectively [33].

In a hospital-based CHB and CHC treatment cost study evaluating 284 patients in the Denizli province in Turkey, it was found that the treatment cost and total cost were higher with the Entecavir treatment compared to the Tenofovir and Lamivudine treatments. PEG Int-2a and 2b treatments had a higher cost than Lamivudine, Entecavir, Tenofovir treatments, and no treatment [34].

In an analysis including the US, Germany, and some Asian countries conducted by Lui et al. [35], the most cost-effective treatment was found to be Entecavir and Tenofovir monotherapies among switching from Telbivudine and Lamivudine monotherapies to Tenofovir and Entecavir treatments.

In other studies, Tenofovir was reported to be the most cost-effective treatment among Tenofovir, Entecavir, Telbivudine, and Adefovir treatments [35, 36].

There are limitations of this study as assumptions. All assumptions and articles that were used in the study were taken from three experts’ opinions.

In the model presented in this study used for the cost-effectiveness analysis of CHB treatments, Tenofovir was found to be one of the cost-effective methods in comparison with other treatment strategies at different time intervals. In addition to this achievement, Tenofovir has been shown to reduce the cumulative treatment cost in the first line of CHB treatment when compared to a 40-year cumulative treatment cost.

In addition, the sensitivity analysis showed that the most effective parameter is the virologic respond; however, TDF had the lowest cost and the highest effectiveness rates as a cost-effective option in all scenarios.

In conclusion, it could be stated that Tenofovir provides cost-effective results regarding public costs and sustainable health financing by being included in the reimbursement for the first line of CHB treatment both in less years of life lost and the reduced cumulative treatment cost.

## Abbreviations

CHB: chronic hepatitis B
HBV: hepatitis B virus
SUT: Healthcare Implementation Notification
YPLL: Years of Potential Life Lost
ICER: incremental cost-effectiveness ratio
HBsAg: hepatitis B surface antigen
CI: confidence interval
3TC: lamivudine
LdT: telbivudine
ETV: Entecavir
TDF: Tenofovir
ETV-TDF: switching to tenofovir from entecavir
LdT-TDF: switching to tenofovir from telbivudine
3TC-TDF: switching to tenofovir from lamivudine
SSI: Social Security Institution
YLL: years of life lost
